# Preoperative Hemoglobin Discriminates Perioperative Transfusion in Reverse Shoulder Arthroplasty: A Single-Center Preliminary Study with Internal Validation and a Candidate Blood-Preparation Threshold

**DOI:** 10.3390/medicina62081455

**Published:** 2026-07-27

**Authors:** Jaemin Lee, Seung Gyu Yang, Doo Sup Kim

**Affiliations:** Department of Orthopedic Surgery, Wonju College of Medicine, Yonsei University, Wonju 26426, Republic of Korea; megatal12@yonsei.ac.kr (J.L.); sgyang0202@yonsei.ac.kr (S.G.Y.)

**Keywords:** reverse shoulder arthroplasty, blood transfusion, preoperative hemoglobin, patient blood management, decision-curve analysis, type-and-screen

## Abstract

*Background and Objectives*: Perioperative transfusion remains a burden after reverse shoulder arthroplasty (RSA). Yet no RSA-specific tool with internally validated discrimination guides preoperative blood preparation. This single-center preliminary study pursued three aims: to derive and internally validate the discrimination of preoperative hemoglobin for transfusion in a protocol-uniform RSA cohort, to characterize its net benefit by decision-curve analysis, and to translate that discrimination into a candidate blood-preparation threshold. *Materials and Methods*: This retrospective study analyzed 214 consecutive elective degenerative primary RSA procedures by a single surgeon. Each was managed with maximal intravenous iron and without routine tranexamic acid. The primary outcome was perioperative transfusion. The analysis fitted Firth penalized models to 213 procedures that included 17 transfusion events. The area under the curve (AUC) quantified preoperative-hemoglobin discrimination, corrected for optimism by bootstrapping, and decision-curve analysis quantified net benefit. *Results*: Transfusion occurred in 7.9% of procedures. Lower preoperative hemoglobin was the prespecified primary predictor, with an adjusted odds ratio of 0.37 per +1 g/dL. As a univariable continuous predictor, it discriminated transfusion with an AUC of 0.867. Internal bootstrap validation showed negligible optimism. Decision-curve analysis showed in-sample net benefit, though only a narrow margin over crossmatch-for-all at the lowest thresholds. At the in-sample Youden cutoff, a hemoglobin above 11.4 g/dL identified about two-thirds of patients. Among them, 1.4% were transfused, for a negative predictive value of 0.99. *Conclusions*: Preoperative hemoglobin showed internally validated discrimination for transfusion. The 11.4 g/dL threshold and its net benefit are apparent (in-sample) and remain fragile given 17 events. Together they define a candidate RSA blood-preparation triage. Because the protocol withheld tranexamic acid, the threshold does not generalize to tranexamic-acid-based practice. These are preliminary, single-center findings. External multicenter validation in larger cohorts is required before adoption.

## 1. Introduction

Reverse shoulder arthroplasty (RSA) has expanded rapidly. Its indications now span cuff tear arthropathy, massive irreparable rotator cuff tear, primary glenohumeral osteoarthritis, and complex proximal humeral fractures. Nationwide data illustrate this growth. In South Korea, total shoulder arthroplasty volume rose almost seven-fold between 2008 and 2017—from 513 to 3583 procedures per year—with reverse arthroplasty driving most of this increase [1]. The most recent national-registry data confirm that the rise continues: the United Kingdom’s National Joint Registry recorded 8221 shoulder arthroplasties in 2023 [2]. As procedural volumes climb in an aging population, perioperative blood loss and allogeneic transfusion remain clinically relevant sources of morbidity, cost, and prolonged hospitalization. Transfused shoulder-arthroplasty patients show higher complications and 90-day mortality, although confounding by indication cannot be excluded [3]. Perioperative blood loss is multifactorial. Surgical and technical determinants each contribute, including longer operative time, humeral stem design, and closed-suction drainage [4,5,6]. Comorbidities and coagulation disorders amplify bleeding further—chronic antiplatelet or anticoagulant therapy [7,8], an elevated preoperative international normalized ratio [9], chronic liver disease [10], and inherited bleeding diatheses [11]. Among patient-level factors, low preoperative hemoglobin nonetheless remains the most consistent and modifiable driver. It is an independent transfusion risk factor in shoulder arthroplasty [12] and shows a graded relationship between anemia severity and transfusion [3,13], with iron-deficiency anemia carrying a markedly elevated risk [14]. Because preoperative hemoglobin dominates transfusion risk, it also anchors two upstream decisions. The first is how blood is prepared, through ordering of type-and-screen versus more resource-intensive type-and-crossmatch. The second is how anemia is corrected, through patient-blood-management programs that include preoperative intravenous (IV) iron [15] and intraoperative tranexamic acid (TXA). TXA reliably reduces blood loss in shoulder arthroplasty [16] yet has not significantly reduced the already low transfusion rate in pooled analysis [17].

Rationalizing blood ordering from the same hemoglobin signal is already established in lower-extremity arthroplasty. There, hemoglobin-based selection spares universal type-and-screen [18], and validated transfusion-prediction models exist for total knee and total hip arthroplasty [19,20]. No comparable blood-preparation tool exists for RSA. A systematic review catalogued eleven shoulder-arthroplasty risk-stratification tools; none predicts transfusion, and anemia enters only a minority, and only as a binary flag [21]. Where preoperative hemoglobin has been used to model shoulder-arthroplasty transfusion, the studies drew on mixed, multi-indication cohorts that pooled anatomic and reverse procedures with high-bleeding fracture and infection cases, and they lacked internal validation [12]. Another shoulder-arthroplasty model reached its strongest discrimination only by adding first-postoperative-day hemoglobin—unavailable for preoperative preparation—to the preoperative value [22]. A frailty index has, in turn, been associated with transfusion after revision shoulder arthroplasty [23]. Most predictor studies draw on administrative or registry data that pool anatomic and reverse arthroplasty [24] and seldom hold the blood-management protocol constant. Heterogeneous, largely unmeasured TXA and IV-iron exposure therefore confounds the reported associations.

The present study addresses this gap in a consecutive single-surgeon RSA cohort. The cohort comprised only elective degenerative indications, so that the bleeding heterogeneity of fracture and trauma would not contaminate patient-level signals. A uniform maximal IV-iron protocol governed care—500 mg of ferric carboxymaltose preoperatively plus 1000 mg on the operative day—without routine TXA. This design holds the principal pharmacologic confounders of perioperative bleeding constant. To date, no other RSA-specific study has united a protocol-uniform pathway with internal validation and an explicit candidate blood-preparation threshold. That combination distinguishes it from prior mixed-indication, heterogeneously managed shoulder-arthroplasty analyses, which neither held the blood-management protocol constant nor internally validated their estimates.

This study therefore pursued three objectives: to derive and internally validate a preoperative-hemoglobin model for perioperative transfusion in elective degenerative RSA, to characterize its net benefit by decision-curve analysis, and to translate it into a candidate blood-preparation triage threshold. The primary hypothesis held that, under a uniform maximal IV-iron, TXA-free protocol, lower preoperative hemoglobin would independently discriminate patients requiring transfusion and would identify a threshold above which most patients could be prepared with type-and-screen alone. The single-center design and the small number of transfusion events temper these aims. The work is therefore best regarded as a preliminary, hypothesis-generating study, intended to motivate external validation rather than to establish a rule ready for adoption.

## 2. Materials and Methods

### 2.1. Study Design

This retrospective, single-center cohort study analyzed consecutive patients who underwent primary RSA at a tertiary referral hospital between 1 January 2018 and 31 December 2025. A single fellowship-trained shoulder surgeon performed every procedure. The report follows the Strengthening the Reporting of Observational Studies in Epidemiology (STROBE) recommendations for cohort studies [25]. The Institutional Review Board of Wonju Severance Christian Hospital, Yonsei University Wonju College of Medicine, approved the protocol (IRB No. CR326065; approved 26 May 2026). The board waived written informed consent, given the retrospective design and the exclusive use of de-identified electronic medical record data. The study followed the Declaration of Helsinki.

### 2.2. Participants

A prospectively maintained operative registry identified the participants. Inclusion criteria were (i) primary RSA performed for an elective degenerative indication—cuff tear arthropathy, massive irreparable rotator cuff tear without glenohumeral arthritis, or primary glenohumeral osteoarthritis—with the degenerative diagnosis confirmed on preoperative radiographs and clinical examination; and (ii) a complete perioperative transfusion record spanning anesthetic induction through hospital discharge. No age or sex restriction was applied. Eligibility rested on the primary indication for arthroplasty rather than on an isolated chart label. A minority of otherwise eligible patients carried a concomitant secondary finding recorded alongside the degenerative primary pathology—such as post-traumatic degenerative change, focal avascular necrosis, a healed fracture sequela, or a coexisting inflammatory or soft-tissue finding. These patients were retained because cuff arthropathy or glenohumeral osteoarthritis remained the basis for the reverse arthroplasty. Exclusion criteria were (i) a primary non-degenerative or non-elective indication—acute proximal humeral fracture, primary post-traumatic or avascular destruction without underlying degenerative or cuff disease, primary inflammatory (e.g., rheumatoid) arthritis, primary or metastatic tumor, or sequelae of prior septic arthritis; (ii) revision of a previously implanted shoulder arthroplasty; and (iii) intraoperative conversion from planned RSA to anatomic total shoulder arthroplasty (TSA).

In total, 218 procedures planned as elective degenerative primary RSA were screened. Chart and operative-note re-review excluded four, because the planned RSA was converted intraoperatively to anatomic TSA, leaving 214 procedures. Patients with bilateral staged RSA contributed each side as a separate surgical event, so the surgical procedure was the unit of analysis. The de-identified dataset retained no patient identifier linking staged procedures, so within-patient clustering could not be assessed.

### 2.3. Perioperative Blood-Management Protocol

A single institutional pathway governed every case. It combined maximal IV iron with a restrictive transfusion indication and deliberate non-use of routine TXA. The IV iron regimen used ferric carboxymaltose: 500 mg approximately one week before surgery and 1000 mg postoperatively on the day of surgery, for a cumulative 1500 mg per patient. This cumulative dose exceeds that of single-dose preoperative regimens and reflects structured IV-iron use in arthroplasty and hip-fracture programs [15,26,27]. The present report terms this a maximal perioperative IV iron protocol. Preoperative anemia and iron deficiency are common in these candidates and are linked to transfusion [14,28].

The protocol did not administer IV, intra-articular, or topical TXA routinely. This conservative, case-by-case policy departs from the contemporary trend toward routine intraoperative TXA. For that trend, randomized-trial meta-analyses demonstrate reduced perioperative blood loss [17,29] with only a non-significant trend toward fewer transfusions [29]. One patient received TXA because chronic antiplatelet therapy could not be withheld perioperatively—a circumstance independently associated with increased RSA bleeding [7]. The analysis therefore treated this TXA exposure descriptively rather than as an analyzed factor. This uniform maximal-iron, TXA-free protocol is the setting in which the candidate blood-preparation tool was developed [18].

Allogeneic packed red blood cell (PRBC) transfusion was considered when any of the following occurred: a hemoglobin of 8.0 g/dL or lower on any perioperative measurement, a postoperative hypotensive (shock) event, or severe anemia-related symptoms. The 8.0 g/dL level served as the principal laboratory trigger rather than an automatic mandate, integrated with hemodynamic and symptomatic status and anticipated recovery under IV iron. Each indication prompted one unit of leukoreduced PRBCs, with reassessment before any further unit. This restrictive threshold falls within the 7–8 g/dL range commonly applied to stable surgical patients.

### 2.4. Surgical Technique

Every patient underwent the same operative technique. The same surgeon performed all procedures through a standardized deltopectoral approach in the beach-chair position, with a mechanical limb-positioning device under general anesthesia. When a repairable subscapularis tendon was present, it was released by a tenotomy approximately 1 cm medial to the bicipital groove and reattached at closure; when no viable subscapularis remained, as in cuff-deficient shoulders, no repair was performed. After glenoid exposure and reaming, a baseplate was secured with central and peripheral screw fixation and a glenosphere was impacted; the humeral component was then implanted, without cement in the large majority of cases (205 of 214) and cemented in the remaining nine. The reverse systems used were Exactech, Zimmer Biomet, DePuy, and Implantcast (Table 1). Intravenous cefazolin (2 g within 60 min before skin incision, continued for approximately 24 h postoperatively) provided antibiotic prophylaxis; consistent with the low venous-thromboembolism risk of upper-extremity arthroplasty and a policy of early postoperative mobilization, no routine pharmacologic thromboprophylaxis was administered. Conventional monopolar electrocautery alone achieved intraoperative hemostasis in every case. No procedure used bipolar electrocautery, topical anti-hemorrhagic or hemostatic agents (topical thrombin, fibrin sealant, gelatin–thrombin matrix, oxidized regenerated cellulose, or topical tranexamic acid), or advanced local-hemostasis devices such as a bipolar radiofrequency sealer, which kept the intraoperative hemostatic technique uniform across the cohort. A closed-suction drain was placed in every case and removed on the second postoperative day—a practice associated with greater hemoglobin decline in shoulder arthroplasty without fewer complications [6]. Admission occurred the day before surgery. The surgical approach, operative sequence, subscapularis and glenoid management, and hemostatic technique did not vary across the cohort, whereas the implant system did (Table 1).

### 2.5. Outcomes and Variables

The prespecified primary outcome was perioperative allogeneic transfusion, defined as administration of any allogeneic PRBC unit from induction of anesthesia through discharge and analyzed as a binary outcome. Secondary outcomes were the number of PRBC units, the perioperative hematologic trajectory (preoperative hemoglobin, postoperative nadir hemoglobin, and the absolute decline), and the intraoperative estimated blood loss.

The abstracted variables spanned three groups. The first comprised age, sex, body mass index (BMI), American Society of Anesthesiologists physical status, primary diagnosis, operative side, and bleeding-relevant comorbidities (hypertension, diabetes mellitus, chronic kidney disease, cardiovascular disease, and malignancy). The second comprised anesthesia type and duration, operative time, and estimated blood loss. The third comprised the hematologic variables. The preoperative hemoglobin was the most recent value before surgery. This value generally followed the preoperative ferric carboxymaltose dose and therefore reflected the iron-treated rather than an untreated baseline. All hemoglobin values came from the institution’s single central-laboratory automated analyzer, and preoperative anemia followed World Health Organization (WHO) sex-specific criteria (hemoglobin <13 g/dL for men, <12 g/dL for women). White-cell count, estimated glomerular filtration rate, serum albumin, intraoperative fluids, implant details, and humeral fixation method were recorded but not entered as candidate predictors, consistent with the parsimonious modeling imposed by the low event count. Chronic kidney disease served as the renal-risk descriptor in Table 1.

### 2.6. Sample Size

This retrospective study enrolled every consecutive eligible patient from the fixed single-surgeon operative census. The available clinical experience therefore governed the sample size rather than a prospective choice, and a formal a priori sample-size calculation was not applicable. Enrolling the complete consecutive series, rather than a sampled subset, also avoided the selection bias that subsampling could introduce. Sample adequacy was instead evaluated after analysis from the number of transfusion events, the events-per-variable (EPV) ratio, and the width of each 95% confidence interval (CI). The modest event count is acknowledged as a limitation and is the explicit rationale for the parsimonious, penalized modeling detailed below.

The adequacy of the realized sample for the single confirmatory analysis—the discrimination of preoperative hemoglobin—was quantified after the fact along three axes. First, that confirmatory analysis used a single predictor and therefore carried 17 events per variable, above the conventional target of about 10. Second, rather than the uninformative power computed at the observed effect, the minimum detectable effect was estimated by simulation (2000 replications, two-sided α = 0.05, with the marginal transfusion probability held at the observed rate): the cohort had at least 80% power to detect an odds ratio of 0.61 per 1 g/dL and 90% power to detect 0.56, both considerably weaker than the observed association of 0.37 per 1 g/dL. Third, the realized precision was correspondingly narrow, the discrimination being estimated with a 95% CI half-width of 0.07 for the area under the curve. These metrics indicate that the sample was sufficient for the primary discrimination analysis. The trivariable estimates and the in-sample 11.4 g/dL threshold, by contrast, rest on the same modest number of events (EPV, 5.7) and are therefore reported as hypothesis-generating rather than as adequately powered estimates—the explicit reason confirmatory inference is restricted to preoperative hemoglobin.

### 2.7. Statistical Methods

Distributional inspection and the Anderson–Darling (n ≥ 50) or Shapiro–Wilk (n < 50) test guided the presentation of continuous variables as mean ± standard deviation when approximately normal and as median [interquartile range] otherwise. Categorical variables appear as n (%). Proportions, including the transfusion rate and operating-characteristic metrics, are reported with Wilson 95% CIs. The Mann–Whitney U test and the χ^2^ or Fisher exact test compared transfused and non-transfused patients. These two groups are defined by the outcome rather than randomly allocated, so baseline differences between them—for example, in age—are expected. Such differences were treated as descriptive screening rather than the basis of inference, and any imbalance was addressed by multivariable adjustment in the primary model.

The primary inferential analysis was a logistic regression model for perioperative transfusion. This model served as the associational basis for the preoperative-hemoglobin-guided tool. Because transfusion was uncommon, the analysis prespecified a parsimonious covariate set: preoperative hemoglobin, age, and sex, with BMI added only if the event count permitted. Firth penalized logistic regression then fitted these covariates, correcting small-sample bias and possible separation. Preoperative hemoglobin was the lead predictor because it is consistently dominant in lower-extremity arthroplasty models [30,31]. The two groups differed in age at baseline, so age was retained as a prespecified adjustment covariate, allowing the hemoglobin association to be estimated net of age. With 17 events, the EPV for the three-covariate model was 5.7, below the conventional target of about 10. This shortfall was the rationale for Firth penalization and for restricting confirmatory inference to preoperative hemoglobin, with other estimates provisional. The remaining clinically relevant variables—anesthesia duration and the comorbidities—were compared between groups (Table 1) rather than entered into a saturated model. Firth-adjusted odds ratios (ORs) are reported with profile penalized-likelihood 95% CIs and Wald z-test *p*-values; the hemoglobin effect is expressed per +1 g/dL, and age and hemoglobin were modeled as linear terms. Other predictors and bivariate comparisons were interpreted descriptively, with *p*-values uncorrected for multiplicity. Because intraoperative blood loss and intraoperative transfusion lie partly on the causal pathway, the primary model was restricted to preoperative and baseline-surgical covariates. An intraoperative-augmented model is reported only as a secondary sensitivity analysis.

A prespecified secondary analysis quantified the discrimination of preoperative hemoglobin by the area under the receiver-operating-characteristic curve (AUC) with a DeLong 95% CI. The Youden J statistic identified a candidate threshold, reported with sensitivity, specificity, and predictive values (Wilson 95% CIs)—an approach previously applied in elective hip arthroplasty [32]. Any such cutoff is derived in-sample, so it was interpreted as a working threshold still requiring external validation. Harrell bootstrap optimism correction (1000 resamples) assessed internal validity, refitting the Firth model in each sample and reporting the optimism-corrected AUC and calibration slope. This bootstrap correction and the decision-curve analysis used the univariable preoperative-hemoglobin Firth model, distinct from the trivariable primary model. Decision-curve analysis quantified the apparent (in-sample) net benefit across the clinically relevant threshold-probability range, compared with preparing crossmatched blood for all patients and for none [20]; these estimates were not optimism-corrected. As an exploratory translation, a candidate blood-preparation rule applied the Youden cutoff: type-and-crossmatch for hemoglobin ≤11.4 g/dL, type-and-screen otherwise. For each arm, the tabulation reported the number of patients, the number and rate of transfusions, and the negative predictive value [18,21].

Prespecified sensitivity analyses comprised preoperative anemia (binary, WHO) in place of continuous hemoglobin, exclusion of the single TXA-exception patient, addition of intraoperative blood loss, addition of BMI, and a univariable preoperative-hemoglobin model. A post hoc exploration characterized the transfusions occurring above the triage threshold and tested whether adding a comorbidity override (chronic kidney disease, or age ≥ 85 years) to the hemoglobin trigger would recover them. All tests were two-sided with α = 0.05.

### 2.8. Missing Data

One of the 214 procedures—a non-transfused patient—lacked a preoperative hemoglobin and was excluded from the preoperative-hemoglobin-based analyses. Those analyses therefore drew on a complete-case sample of 213 procedures with 17 transfusion events. Inconsistent laboratory date stamps prevented reliable dating of the postoperative nadir in 22 of these 213 procedures, so this measure is summarized descriptively for 191 procedures (Table 2); it affected no inferential analysis. No other variable had material missingness beyond the per-variable denominators reported in Table 1; the completeness of every analytic variable is summarized in Table S2.

### 2.9. Bias

Several steps limited bias. Patients were enrolled consecutively from the prospectively updated registry, the transfusion outcome was ascertained from the objective hospital blood-bank ledger, and all hemoglobin values were measured on a single central-laboratory analyzer.

### 2.10. Software

Analyses used Python 3.11.9 with pandas 3.0.2, statsmodels 0.14.6, scikit-learn 1.8.0, scipy 1.17.1, and matplotlib 3.10.8. The bootstrap optimism correction and decision-curve net-benefit analysis used custom scripts.

### 2.11. AI Statement

Generative artificial intelligence (GenAI) was used only as a language, organizational, and analysis-support tool; it contributed no study data and no clinical content. Claude Code (Anthropic, version 2.1) assisted with English-language editing, with organizing the structure of the manuscript, with curating and formatting the reference library, and with writing and running the Python 3.11.9 scripts that executed the prespecified statistical analyses and produced the associated figures. No GenAI tool was used to design the study, to collect, generate, or alter clinical data, or to interpret the findings; every statistical result reported here was produced by the analysis code running on the study dataset and was checked by the authors against the source data. The authors reviewed and verified every AI-assisted output and take full responsibility for the content of the manuscript; no AI tool meets authorship criteria, and none is listed as an author.

## 3. Results

### 3.1. Patient Flow and Characteristics

**Figure 1:**
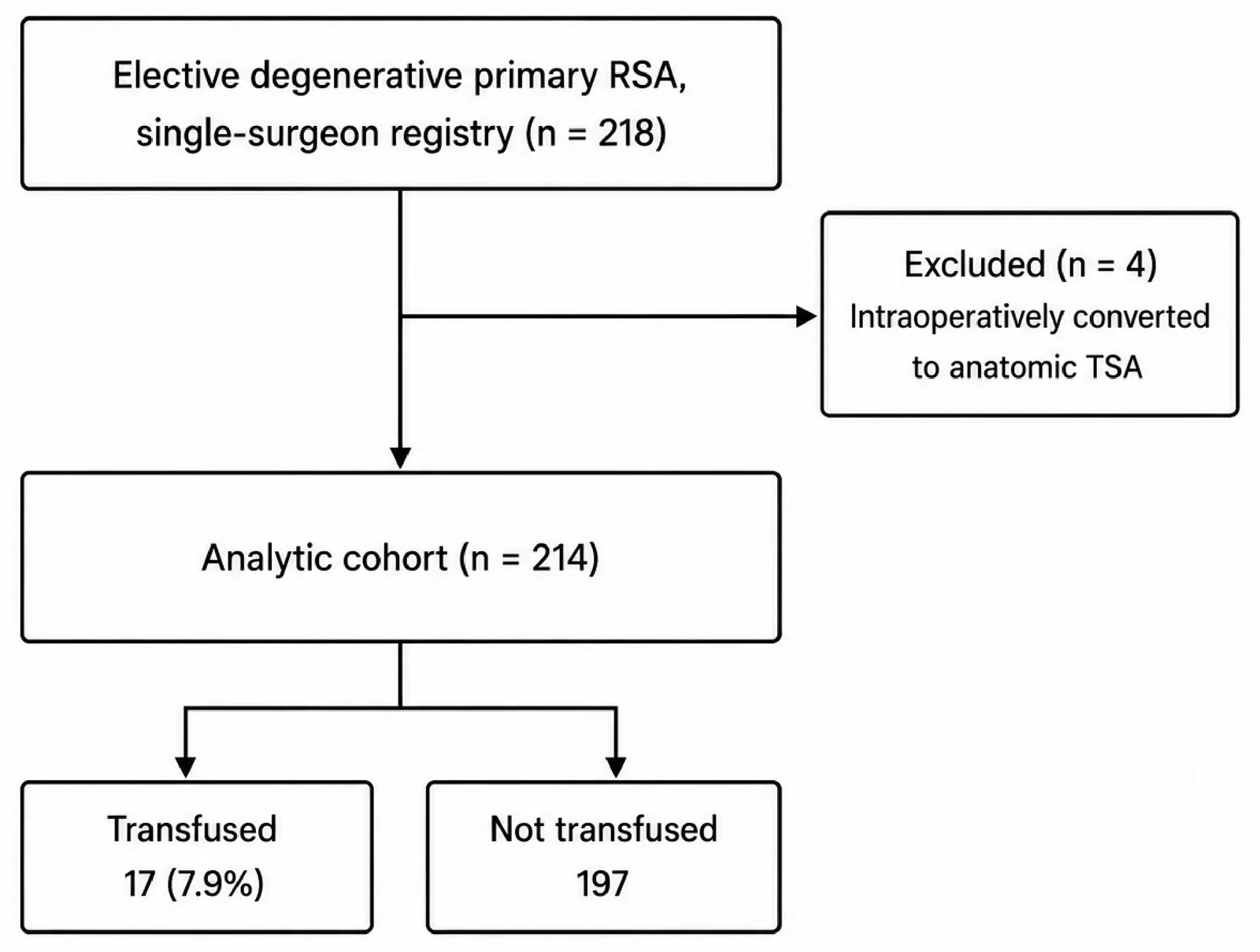
Patient flow diagram. Of 218 procedures planned as elective degenerative primary RSA, four were excluded for intraoperative conversion to anatomic TSA, leaving 214 analytic procedures; hemoglobin-based analyses included 213, because one non-transfused patient lacked a preoperative hemoglobin. RSA, reverse shoulder arthroplasty; TSA, total shoulder arthroplasty.

Elective degenerative primary RSA was planned for 218 procedures. Four were excluded for intraoperative conversion to anatomic TSA, leaving 214 primary RSA procedures (Figure 1). All 214 carried complete blood-bank transfusion records through discharge. Table 1 presents the operative indications and baseline characteristics, both overall and by transfusion status.

### 3.2. Primary Outcome—Perioperative Transfusion

Perioperative allogeneic transfusion occurred in 17 of 214 procedures (7.9%). Sixteen of the 17 transfused patients were anemic before surgery. Table 2 summarizes the hematologic and transfusion outcomes.

### 3.3. Predictors of Transfusion

The Firth penalized model was fitted on the 213 procedures with a complete preoperative hemoglobin. Lower preoperative hemoglobin was the prespecified predictor of transfusion. Older age showed a secondary association, whereas female sex did not (Table 3). The prespecified sensitivity analyses were directionally concordant with the primary model (Table S1).

### 3.4. Discrimination of Preoperative Hemoglobin

**Figure 2:**
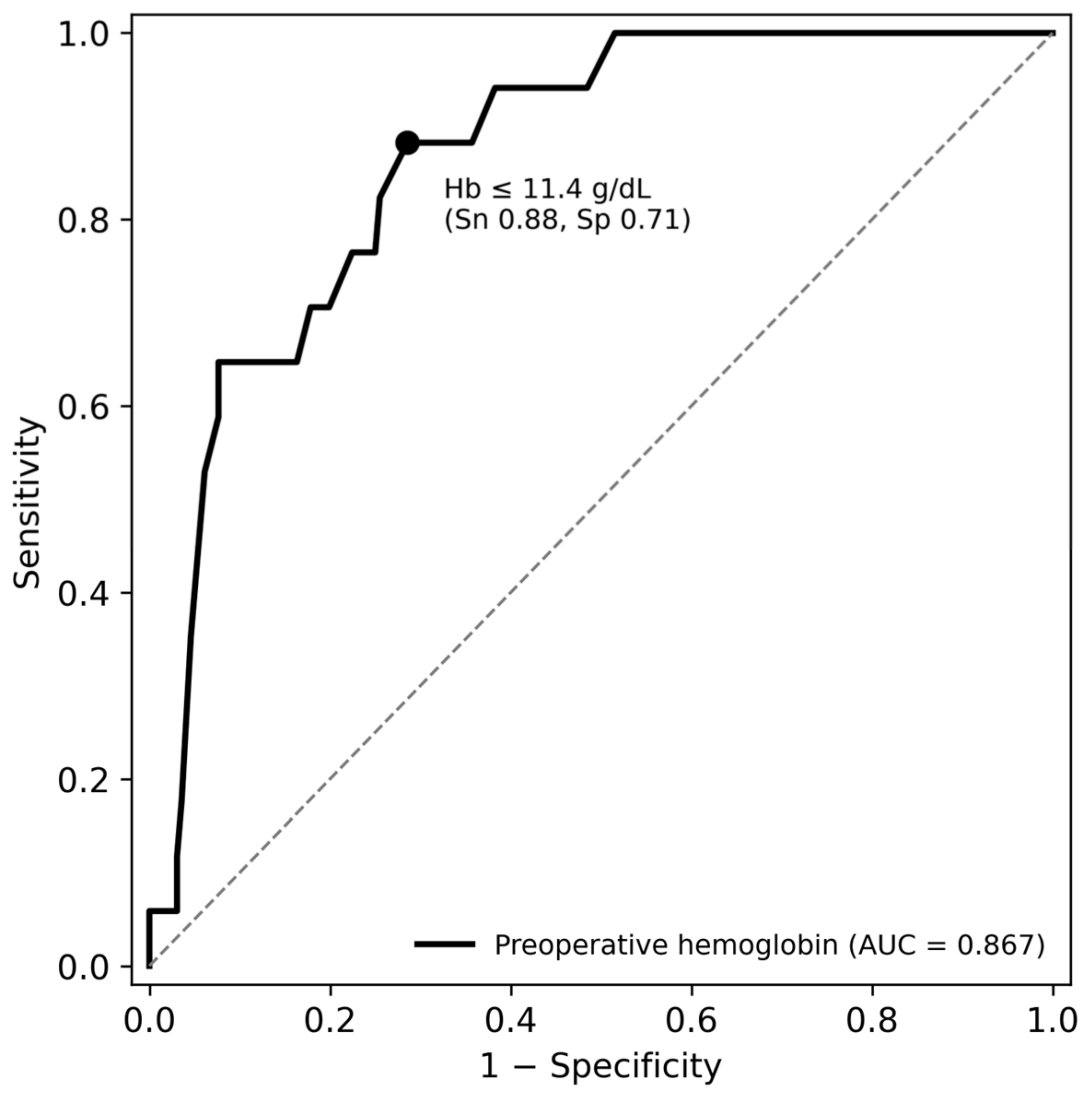
Receiver-operating-characteristic curve of preoperative hemoglobin for perioperative transfusion. Discrimination of perioperative transfusion by preoperative hemoglobin; area under the curve (AUC), 0.867. The dashed diagonal is the reference line of no discrimination (AUC = 0.50), and the filled circle marks the Youden-optimal operating point; the marked cutoff of 11.4 g/dL is hypothesis-generating.

Preoperative hemoglobin discriminated perioperative transfusion (Figure 2). Table 4 reports the area under the curve, the Youden-optimal cutoff, and the operating characteristics.

### 3.5. Internal Validation and Decision-Curve Analysis

**Figure 3:**
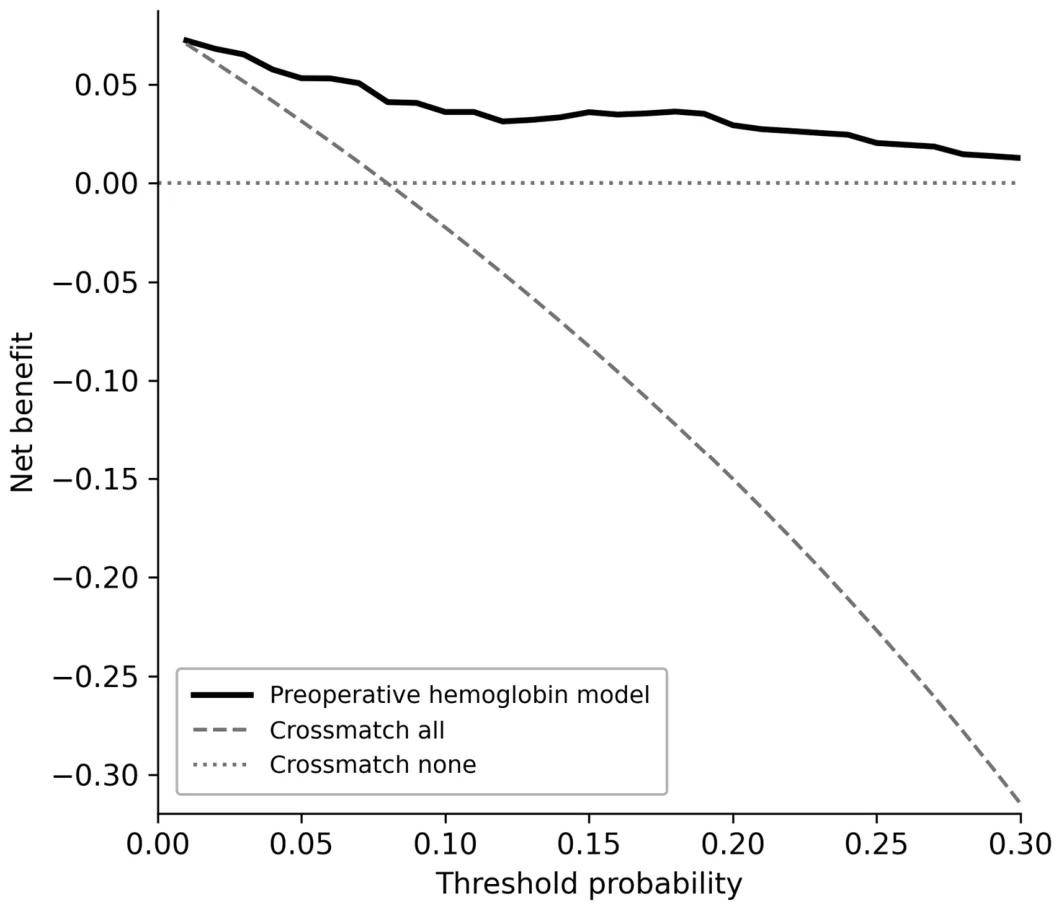
Decision-curve analysis of preoperative hemoglobin-guided blood preparation. Net benefit of the preoperative-hemoglobin model, compared with the crossmatch-for-all and crossmatch-for-none strategies; net benefit exceeded both across the 1–30% threshold range, with a narrow margin over crossmatch-for-all at the lowest thresholds. These estimates are apparent, in-sample, and not optimism-corrected.

Table 4 also presents internal validation by Harrell optimism correction (1000 bootstrap resamples). On decision-curve analysis, the univariable preoperative-hemoglobin model showed positive net benefit across the 1–30% threshold-probability range. This net benefit exceeded both the crossmatch-for-all and crossmatch-for-none strategies (Figure 3). These net-benefit estimates were apparent (in-sample) and not optimism-corrected. Table 5 reports the candidate blood-preparation triage applied at the Youden-optimal cutoff.

## 4. Discussion

### 4.1. Main Findings

This consecutive single-surgeon cohort comprised 214 primary RSAs managed with maximal perioperative IV iron and without routine TXA. Perioperative transfusion was uncommon (7.9%), and its rate tracked the preoperative hematologic reserve. Lower preoperative hemoglobin was the prespecified confirmatory predictor, and older age a secondary, exploratory association; female sex was not associated with transfusion (Table 3). Preoperative hemoglobin discriminated transfusion (AUC, 0.867; Table 4). Most transfused patients were anemic despite pharmacologic pre-treatment and removal of the antifibrinolytic confounder. These relationships are associational. The design identifies who is transfused, not why.

The contribution is not the well-established fact that preoperative hemoglobin predicts transfusion. Rather, under a protocol-uniform pathway, this dominant, internally validated predictor can anchor a candidate rule for triaging blood preparation before RSA—a use for which no shoulder-arthroplasty tool currently exists. The cutoff and triage still require external validation.

### 4.2. Comparison with the Literature

The observed 7.9% rate is best read against RSA-specific benchmarks rather than pooled estimates from heterogeneously managed cohorts. Contemporary RSA series span a wide band. A drain-based study reported 2.6% within a cited range of 2–11% [33]. A stemless-versus-stemmed comparison reported 0% versus 6.7% [5]. A topical-TXA cohort reported roughly 7.7% versus 6.8% [34]. In a national database, the reverse-arthroplasty arm for proximal humerus fracture carried 11.7%, and transfusion was the only complication remaining independently associated with reverse arthroplasty after adjustment [35]. Against this spread, 7.9% in an elective degenerative, TXA-sparing cohort is unremarkable.

The contrast with TXA frames the present protocol choice. TXA reliably reduces perioperative blood loss, but its transfusion effect is mixed. A shoulder meta-analysis found a non-significant effect [17]. A trial-sequential analysis judged the evidence inconclusive [36]. A randomized RSA/TSA trial cut drain volume and hemoglobin drop yet recorded zero transfusions in either arm [16]. Other work still reports a benefit, most notably in fracture patients [37,38]. In a large RSA-inclusive series, the absence of perioperative TXA was the single strongest transfusion risk factor at an overall rate of 16.3%. In that series, abnormal preoperative hemoglobin predicted postoperative anemia but not transfusion [39]. By holding TXA constant and modeling hemoglobin continuously rather than dichotomously, the present design lets the preoperative-hemoglobin signal emerge as the leading correlate. The cohorts nonetheless differ in surgeon number, era, and case mix, so the lower rate cannot be attributed to iron alone. TXA’s hemoglobin-sparing effect in the shoulder is modest (≈ 0.5–0.7 g/dL), and its supporting trials are statistically fragile [29,40]; the shoulder review literature frequently exhibits reporting bias [41]. Withholding it therefore removed a confounder of uncertain marginal benefit.

Beyond antifibrinolytics, a broader hemostatic armamentarium can reduce arthroplasty blood loss, and the deliberately minimal protocol of this study used none of it. Topical hemostatic agents applied to the surgical field—thrombin, fibrin sealants, and gelatin- or cellulose-based matrices—lower blood loss and transfusion in total joint arthroplasty. A meta-analysis of oxidized regenerated cellulose in arthroplasty found reduced total blood loss and hemoglobin drop without added thrombotic or wound-healing risk [42]. In total shoulder arthroplasty, topical thrombin reduced blood loss comparably to intravenous TXA and offers an alternative when antifibrinolytics are contraindicated [43]. Intravenous and topical TXA likewise lower shoulder-arthroplasty blood loss, with no clear advantage of one route over the other [44,45,46]. Meticulous intraoperative coagulation is the complementary lever, and comparisons of advanced local-hemostasis devices with standard electrocautery are mixed. A bipolar sealer reduced transfusion and total blood loss in total hip arthroplasty [47] but yielded only a small blood-loss reduction and no transfusion benefit in total knee arthroplasty [48]. This cohort used conventional monopolar electrocautery alone, without topical agents or a bipolar sealer. That choice serves the study’s aim of isolating the preoperative-hemoglobin signal from pharmacologic and device confounders. It also means the observed 7.9% transfusion rate and the 11.4 g/dL threshold represent a conservative, upper-bound scenario. Centers that routinely add TXA, a topical hemostat, or advanced local coagulation would expect a lower transfusion rate and would need to recalibrate the threshold and its net benefit accordingly. Equally, these adjuncts form a ready menu for intensifying blood conservation in the low-hemoglobin subgroup that the triage rule flags.

What dominated instead was preoperative anemia, the leading independent transfusion risk factor in shoulder-arthroplasty cohorts [12]. Part of its signal may nonetheless reflect frailty or comorbidity rather than a direct causal effect [49]. Iron-deficiency anemia carried a transfusion odds ratio above 10 [14]. Anemia severity scaled steeply with transfusion [3,13]. Each gram per deciliter of hemoglobin lowered transfusion odds in the knee, where anemia became a *greater* predictor over a decade as overall transfusion fell [50,51]. Chronic kidney disease was associated with higher transfusion odds in the shoulder [52] and independently in a national reverse-arthroplasty series [53]. The higher unadjusted proportion among the transfused patients (Table 1) is directionally consistent but rests on few events. Patient-blood-management programs raise hemoglobin, the preoperative lever this protocol pulls, and their adherence is associated with fewer complications, though neither isolates IV iron [15,54].

A large mixed shoulder-arthroplasty series found preoperative anemia significant but not dominant and attributed the attenuated signal to hemoglobin optimization [55]. In this cohort, hemoglobin remained dominant even under maximal IV iron. Optimization therefore appears to shift the distribution upward without erasing the risk in its lowest tail. Age was not independent once hemoglobin was modeled in several lower-extremity series [18,51], though an RSA national series identified advanced age [53]. In this older, predominantly female population (median age of 78), age showed only an exploratory association. The non-dominance of sex once hemoglobin was modeled is concordant with the female signal operating largely through women’s lower baseline reserve [24,56], and the non-dominance of body mass index with the broader literature [57].

The blood-ordering question this raises is already being answered in lower-extremity arthroplasty. There, preoperative hemoglobin has been used to spare universal type-and-screen [18], and validated transfusion-prediction models inform the same decision [19,20]. The shoulder has lagged. In a systematic review of risk-stratification tools, none of the eleven identified instruments predicted transfusion [21]. The present work transplants this logic into RSA with two differences that matter for a triage rule: a uniform IV-iron, TXA-free protocol that removes the antifibrinolytic confounder, and internal validation of the discrimination before any clinical claim.

### 4.3. Clinical Implications

Preoperative hemoglobin is measured before incision, and here it stratified patients by transfusion risk under the IV-iron protocol (Table 4). This makes it a candidate basis for triaging blood preparation rather than merely a risk marker. At the Youden cutoff (11.4 g/dL), sensitivity was high and the negative predictive value was 0.99. That value is buoyed by the low (7.9%) baseline transfusion rate and is expected to attenuate at higher transfusion prevalence. A hemoglobin above 11.4 g/dL would therefore route roughly two-thirds of patients to type-and-screen alone, reserving type-and-crossmatch—and intensified optimization and counseling—for the lower-hemoglobin group. This mirrors the hemoglobin-based selection proposed for total knee arthroplasty under routine TXA [18]. No shoulder-arthroplasty tool currently offers such a rule [21,23]. This RSA-specific, protocol-uniform candidate rule is the first to fill that gap. A similar threshold (≈11.5 g/dL) was previously derived in a predominantly reverse but mixed-indication cohort that also omitted routine TXA [12]. The agreement is reassuring, although that model pooled high-bleeding fracture and infection cases, was not internally validated, and was not framed as a blood-preparation rule. Because the cutoff lies below the WHO anemia thresholds, the rule is more selective than anemia-based ordering; it crossmatches roughly one-third of patients rather than the 57.7% meeting WHO criteria (Table 1).

The two transfusions above the threshold occurred at preoperative hemoglobins only marginally above the cutoff (11.7 and 12.2 g/dL). Both involved large perioperative (preoperative-to-nadir) hemoglobin declines (3.3 and 3.8 g/dL, ≥90th percentile)—bleeding not foreseeable from the preoperative value. A post hoc comorbidity override did not help. A chronic-kidney-disease override recovered only one of the two at one extra crossmatch. An age-based override (≥85 years) recovered the same single case only by crossmatching 90 of 213 patients (42%). The other above-threshold transfusion (preoperative hemoglobin of 11.7 g/dL) was recovered by neither. The appropriate safeguard is therefore retained rapid-crossmatch availability for the type-and-screen group rather than a more elaborate preoperative model.

Internal validation showed negligible optimism (Table 4), though this estimate is unstable given 17 events. Decision-curve analysis showed net benefit that exceeded both the crossmatch-for-all and crossmatch-for-none strategies across the 1–30% threshold range (Figure 3), with only a narrow margin over crossmatch-for-all at the lowest thresholds. This net benefit was computed in-sample. Like the cutoff and triage characteristics, it remains apparent and requires external validation. Because most patients fall above the threshold, most would need only a type-and-screen, paralleling the economy reported in the knee [18]. The trade-off is nonetheless explicit. At a positive predictive value of 0.21, most crossmatches would prove precautionary, and because transfusions still occurred above the threshold, the strategy presumes rapid crossmatch availability. Once externally validated, such a protocol-uniform rule could also support blood-management auditing in a field where transfusion practice remains heterogeneous [58].

### 4.4. Limitations

The study is retrospective and confined to a single surgeon and center. This design maximizes protocol uniformity but constrains external validity. The discrimination is internally validated; the cutoff and rule are not. The transfused and non-transfused groups were defined by the outcome rather than randomly allocated, so they differed at baseline—most notably in age—as is expected in an observational cohort. Age was therefore carried as a prespecified adjustment covariate, and preoperative hemoglobin remained the independent predictor net of it, though residual and unmeasured confounding cannot be excluded. Because TXA was withheld by protocol, the rate, predictor estimates, and threshold do not generalize to TXA-based practice. With no internal TXA or no-iron comparator, the contribution of IV iron cannot be isolated. The model rests on 17 events (EPV, 5.7), so estimates for low-prevalence covariates remain provisional. The Youden cutoff is likewise fragile: moving one transfusion across it would change sensitivity from 0.88 to 0.82 or 0.94, because bootstrapping stabilizes the AUC but not the cutoff. The transfusion outcome was decided clinically under a hemoglobin-based trigger and with knowledge of the preoperative hemoglobin. Preoperative and nadir hemoglobin are correlated. Part of the apparent discrimination may therefore reflect this unblinded decision rather than an independent biological signal. Only blinded or fully protocolized external validation can exclude such ascertainment bias. The intraoperative blood-loss field was zero in most cases and under-captured true blood loss, and every case used a routine closed-suction drain that may augment measured hemoglobin loss. Staged bilateral procedures contributed each shoulder separately, leaving residual within-patient correlation. The 2018–2025 span also encompasses secular change and an evolving implant mix; stemless designs, for instance, are associated with lower blood loss [59]. The protocol nonetheless stayed constant throughout. Because the dataset did not retain operative date, the prespecified operative-year adjustment was not possible. Finally, the restriction to elective degenerative primary RSA gives the cohort its homogeneity but omits higher-bleeding fracture and revision populations, so the findings apply only to this population.

### 4.5. Future Directions

External multicenter validation is the priority. A prospective study is planned to confirm whether the 11.4 g/dL threshold, its net-benefit profile, and its negative predictive value hold across surgeons, centers, and case mixes—the prerequisite for any change in blood-ordering practice. In parallel, a prospective trial could add tranexamic acid to the maximal IV iron pathway, targeting the low-hemoglobin subgroup that accounts for nearly all transfusions. This intensification would test whether the crossmatch group can shrink further. Pairing an externally validated rule with this targeted protocol could move RSA blood preparation toward the hemoglobin-guided, resource-sparing blood-ordering economy increasingly reported in lower-extremity arthroplasty [18,19,20].

## 5. Conclusions

In this single-surgeon RSA cohort managed with maximal intravenous iron and without routine tranexamic acid, perioperative transfusion was uncommon (7.9%). Lower preoperative hemoglobin was the dominant predictor (adjusted odds ratio of 0.37 per +1 g/dL), and the model showed internally validated discrimination. As a candidate triage, a preoperative hemoglobin above 11.4 g/dL would direct roughly two-thirds of patients to type-and-screen alone (negative predictive value, 0.99), reserving crossmatch for lower values. The threshold and its net benefit remain in-sample findings from a single center based on only 17 events, so external multicenter validation in larger cohorts is required before this candidate rule enters practice.

## Figures and Tables

**Table 1:** Baseline characteristics, overall and by perioperative transfusion status.

Variable	Overall (N = 214)	Transfused (n = 17)	Not Transfused (n = 197)	*p*
**Demographics**				
Age, years	78.0 [74.0–83.0]	86.0 [81.0–87.0]	78.0 [74.0–82.0]	<0.001
Female sex	141 (65.9)	14 (82.4)	127 (64.5)	0.184
BMI, kg/m^2^	24.8 [22.6–27.1]	22.4 [20.8–27.2]	24.8 [22.8–27.1]	0.187
**Comorbidities**				
Hypertension	140 (65.7)	13 (76.5)	127 (64.8)	0.429
Diabetes mellitus	59 (27.7)	5 (29.4)	54 (27.6)	1.000
Chronic kidney disease	9 (4.2)	4 (23.5)	5 (2.5)	0.003
Cardiovascular disease	54 (25.2)	8 (47.1)	46 (23.4)	0.062
**Anesthesia and surgery**				
General anesthesia	214 (100)	17 (100)	197 (100)	—
Anesthesia duration, min	130.0 [120.0–140.0]	140.0 [135.0–160.0]	126.0 [120.0–140.0]	<0.001
ASA 1/2/3, n	4/98/112	0/3/14	4/95/98	0.035
Implant (Exactech/Zimmer/DePuy/Implantcast)	116/84/9/5	—	—	—
**Baseline hematology**				
Preoperative hemoglobin, g/dL	12.0 [11.0–13.0]	9.9 [9.5–11.0]	12.2 [11.2–13.0]	<0.001
Preoperative anemia (WHO)	123 (57.7)	16 (94.1)	107 (54.6)	0.001
Nadir hemoglobin, g/dL	9.9 [9.1–11.1]	8.0 [7.1–8.4]	10.1 [9.3–11.2]	<0.001
**Blood management**				
Ferric carboxymaltose (500 + 1000 mg)	214 (100)	17 (100)	197 (100)	—
Tranexamic acid administered	1 (0.5)	0 (0)	1 (0.5)	—

Data are median [IQR] or n (%). Continuous variables compared by the Mann–Whitney U test; categorical by the χ^2^ or Fisher exact test. Denominators: BMI, 210; hypertension/diabetes, 213; preoperative hemoglobin/anemia/nadir, 213; others, 214. Abbreviations: ASA, American Society of Anesthesiologists; BMI, body mass index; IQR, interquartile range; WHO, World Health Organization.

**Table 2:** Perioperative hematologic and transfusion outcomes (N = 214).

Outcome	Value (N = 214)
**Transfusion**	
Any perioperative allogeneic transfusion, n (%, 95% CI)	17 (7.9%, 5.0–12.4)
Intraoperative transfusion, n (%)	2 (0.9%)
PRBC units, total	34
PRBC units per patient (whole cohort), mean	0.16
PRBC units among transfused, mean (median)	2.0 (2)
**Hemoglobin trajectory**	
Preoperative hemoglobin, g/dL	12.0 ± 1.5 (median 12.0 [11.0–13.0])
Nadir hemoglobin, g/dL	10.0 ± 1.5 (median 9.9 [9.1–11.1])
Postoperative day of nadir	2 [2–3]
Absolute hemoglobin decline (preop − nadir), g/dL	2.0 ± 1.0 (median 1.9 [1.2–2.5])
Preoperative anemia (WHO), n (%)	123 (57.7%)
**Blood loss and other**	
Intraoperative estimated blood loss, mL	46 ± 153 (median 0 [0–0]) †

Data presented as mean ± SD, median [IQR], or n (% [Wilson 95% CI]). PRBCs, packed red blood cells; WHO, World Health Organization. † Recorded intraoperative blood output was 0 in most cases and right-skewed and likely under-captures true intraoperative blood loss. The postoperative day of nadir is summarized for the 191 procedures with a plausible recorded nadir date; 22 records with inconsistent laboratory date stamps (implying a nadir before surgery or beyond 30 days) were excluded from this descriptive field only. Preoperative hemoglobin, nadir hemoglobin, hemoglobin decline, and preoperative-anemia denominator = 213 (one missing preoperative hemoglobin).

**Table 3:** Firth penalized logistic regression—predictors of perioperative allogeneic transfusion after RSA.

Predictor	Firth-Adjusted OR	95% CI	*p*
Preoperative hemoglobin, per +1 g/dL	0.37	0.21–0.58	<0.001
Age, per +1 year	1.21	1.08–1.39	0.002
Female sex (vs. male)	1.48	0.39–7.15	0.59

Outcome: any perioperative allogeneic PRBC transfusion (17 events among 213 procedures; one patient excluded for a missing preoperative hemoglobin). Model fitted by Firth penalized logistic regression (Jeffreys prior bias correction) given the low event count; the predictor set was pre-specified and capped at three covariates (events-per-variable = 5.7). Confidence intervals are profile penalized-likelihood intervals; *p*-values are Wald-type z-tests from the penalized-likelihood information matrix. Preoperative hemoglobin is the single pre-specified confirmatory predictor; age and sex are secondary with descriptive *p*-values. Body mass index was not added to the primary model to preserve the events-per-variable ratio; in a sensitivity model that added body mass index, the hemoglobin and age estimates were essentially unchanged and body mass index was non-significant (OR 1.04 per kg/m^2^, 95% CI 0.87–1.24, *p* = 0.68). The remaining candidate predictors (ASA class, anesthesia duration, hypertension, diabetes, chronic kidney disease, cardiovascular disease) appear in the Table 1 bivariate comparison. Abbreviations: CI, confidence interval; OR, odds ratio; PRBCs, packed red blood cells; RSA, reverse shoulder arthroplasty.

**Table 4:** Discrimination of preoperative hemoglobin for perioperative transfusion (ROC).

Predictor	AUC	95% CI	Youden Cutoff	Sensitivity	Specificity	PPV	NPV
Preoperative hemoglobin	0.867	0.793–0.940	≤11.4 g/dL	0.88 (0.66–0.97)	0.70 (0.64–0.76)	0.21 (0.13–0.31)	0.99 (0.95–1.00)

Outcome: any perioperative allogeneic transfusion (17 events/213; one patient with a missing preoperative hemoglobin was excluded from this analysis). The area under the receiver-operating-characteristic curve (AUC) 95% CI is the DeLong interval for the apparent AUC (the optimism-corrected AUC, which coincides with the apparent value, is not separately interval-estimated); sensitivity, specificity, PPV, and NPV are shown with Wilson 95% confidence intervals. The Youden-optimal cutoff and its operating characteristics are reported in-sample and are hypothesis-generating only; given 17 events the confidence interval is wide and external validation is required before any threshold is adopted. The high negative predictive value (0.99) reflects the low baseline transfusion rate. Internal validation of the same Firth penalized model by 1000-bootstrap optimism correction gave an optimism-corrected AUC of 0.867 (apparent, 0.867; optimism, −0.001) and a calibration slope of 1.04. The discrimination showed little apparent optimism on internal bootstrap; residual overfitting cannot be excluded, and external validation remains required. The slope slightly exceeds 1.0 because Firth penalization shrinks the coefficient (predictions are marginally under-extreme rather than overfit), and the bootstrap optimism in the slope was negligible (the corrected and apparent slopes coincide). Abbreviations: AUC, area under the receiver-operating-characteristic curve; NPV, negative predictive value; PPV, positive predictive value; ROC, receiver operating characteristic.

**Table 5:** Preoperative hemoglobin-guided blood-preparation triage (cutoff ≤ 11.4 g/dL).

Blood-Preparation Group	n (%)	Transfused, n (%)	Candidate Strategy
Preoperative hemoglobin ≤ 11.4 g/dL	73 (34.3)	15 (20.5)	Type-and-crossmatch
Preoperative hemoglobin > 11.4 g/dL	140 (65.7)	2 (1.4)	Type-and-screen

Applied to the 213 procedures with an available preoperative hemoglobin (17 transfusions). The ≤11.4 g/dL threshold (Youden-optimal; Table 4) captured 15 of 17 transfusions (sensitivity, 0.88) with a negative predictive value of 0.99. A rule reserving type-and-crossmatch for preoperative hemoglobin ≤ 11.4 g/dL and type-and-screen for higher values would direct roughly two-thirds of patients to type-and-screen alone. The underlying continuous model’s discrimination was internally validated (Table 4); the ≤11.4 g/dL threshold itself is in-sample and hypothesis-generating, derived in a single center, and requires external validation before adoption.

## Data Availability

The data are not publicly available because they contain protected health information restricted by the approving Institutional Review Board and by Korean medical-information regulations. The de-identified analytic dataset and analysis scripts are available from the corresponding author on reasonable request and are subject to renewed institutional approval.

## Supplementary Materials

The following supporting information can be downloaded at: https://www.mdpi.com/article/10.3390/medicina62081455/s1, Table S1. Firth penalized logistic regression—primary and sensitivity models; Table S2. Completeness of analytic variables (N = 214).

## References

[1] Park J.S., Lee H.J., Jo Y.H., Lee M.K., Lee B.G. (2023). Surgical Trends of Shoulder Arthroplasty: Nationwide Epidemiologic Study in South Korea. Clin. Orthop. Surg..

[2] O’Malley O., Davies A., Rangan A., Sabharwal S., Reilly P. (2026). Predictors of prolonged length of stay in shoulder arthroplasty: A study using the National Joint Registry and Hospital Episode Statistics for England. Shoulder Elb..

[3] Alder K.D., Yu K.E., Rode M.M., Marigi I.M., Marigi E.M., Morrey M.E., Sperling J.W., Sanchez-Sotelo J. (2024). Increasing severity of preoperative anemia is associated with higher postoperative medical and surgical complications after primary shoulder arthroplasty. J. Shoulder Elb. Surg..

[4] Gruson K.I., Lo Y., Stallone S., Qawasmi F., Lee S., Shah P. (2022). A Comparison of Operative Time and Intraoperative Blood Volume Loss Between Stemless and Short-stem Anatomic Total Shoulder Arthroplasty: A Single Institution’s Experience. JAAOS Glob. Res. Rev..

[5] Sakek F., Haight H., Tuphé P., Regas I., Adam A., Rochet S., Lascar T., Obert L., Loisel F. (2022). Assessment of intraoperative bleeding in reverse shoulder arthroplasty—With or without a stem. Orthop. Traumatol. Surg. Res..

[6] Oliveira Almeida L.H., La Banca V., Matsumoto O., Steven de Sá A., Palagi Viganó A.V., Kalman G., Gonzalez F.F., Lima G.H.V., Murachovsky J., Ikemoto R.Y. (2025). Blood loss, complications, and the role of suction drains in shoulder arthroplasty: A systematic review and meta-analysis. JSES Int..

[7] Yamakado K. (2025). Impact of antiplatelet and anticoagulant drugs on postoperative bleeding in reverse total shoulder arthroplasty. J. Shoulder Elb. Surg..

[8] Mao X., Liang C., Li X., Shi D., Yang Q., Xie H., Liang F., Cui Y. (2023). The impact of long-term aspirin use on the patients undergoing shoulder arthroplasty. J. Orthop. Surg. Res..

[9] Zhang D., Dyer G.S.M., Earp B.E. (2024). The Relationship Between Preoperative International Normalized Ratio and Postoperative Major Bleeding in Total Shoulder Arthroplasty. JAAOS Glob. Res. Rev..

[10] Martino J.A., Guareschi A.S., Rogalski B.L., Eichinger J.K., Friedman R.J. (2026). Cirrhosis associated with increased complications and healthcare utilization following total shoulder arthroplasty. Shoulder Elb..

[11] Gillinov S.M., Modrak M., Park N., Monahan P.F., Wilhelm C.V., Lee M.S., Mahatme R.J., Fong S., Moran J., Grauer J.N. (2025). Total Shoulder Arthroplasty in Patients with Hemophilia A: Greater Odds of Postoperative Bleeding and Thromboembolic Events but No Difference in 5-year Implant Survival. Clin. Orthop. Relat. Res..

[12] Kopechek K.J., Frantz T.L., Everhart J.S., Samade R., Bishop J.Y., Neviaser A.S., Cvetanovich G.L. (2022). Risk factors for postoperative blood transfusion after shoulder arthroplasty. Shoulder Elb..

[13] Wang K.Y., Quan T., Gu A., Best M.J., Stadecker M., Srikumaran U. (2021). Increased severity of anemia is associated with postoperative complications following primary total shoulder arthroplasty. J. Shoulder Elb. Surg..

[14] Polisetty T., Cannon D., Grewal G., Vakharia R.M., Vegas A., Levy J.C. (2023). Iron deficiency anemia is associated with increased medical and implant-related complications and length of stay for patients undergoing total shoulder arthroplasty. J. Shoulder Elb. Surg..

[15] Buddhiraju A., Agarwal S., Ro J., Hegde V., Liu S., Khanuja H.S. (2026). Optimizing Anemia in Total Hip Arthroplasty: Experience with an Institutional Protocol. J. Arthroplast..

[16] Cunningham G., Hughes J., Borner B., Mattern O., Taha M.E., Smith M.M., Young A.A., Cass B. (2021). A single dose of tranexamic acid reduces blood loss after reverse and anatomic shoulder arthroplasty: A randomized controlled trial. J. Shoulder Elb. Surg..

[17] Tan T.K., Tan P., Wang K., Hau R. (2024). Effect of tranexamic acid on shoulder surgery: An updated meta-analysis of randomized studies. J. Shoulder Elb. Surg..

[18] Haider M.A., Habibi A., Ward S.A., Rozell J.C., Macaulay W., Schwarzkopf R., Hepinstall M. (2026). Blood Transfusion in the Age of Tranexamic Acid: Who Needs a Type and Screen Before Total Knee Arthroplasty?. J. Arthroplast..

[19] Kolin D.A., Lyman S., Della Valle A.G., Ast M.P., Landy D.C., Chalmers B.P. (2023). Predicting Postoperative Anemia and Blood Transfusion Following Total Knee Arthroplasty. J. Arthroplast..

[20] Buddhiraju A., Shimizu M.R., Subih M.A., Chen T.L., Seo H.H., Kwon Y.M. (2023). Validation of Machine Learning Model Performance in Predicting Blood Transfusion After Primary and Revision Total Hip Arthroplasty. J. Arthroplast..

[21] Imam N., Sudah S.Y., Manzi J.E., Sirch F., Nicholson A.D., Denard P.J., Menendez M.E. (2023). Perioperative risk stratification tools for shoulder arthroplasty: A systematic review. J. Shoulder Elb. Surg..

[22] Belay E.S., Flamant E., Sugarman B., Goltz D.E., Klifto C.S., Anakwenze O. (2021). Utility of postoperative hemoglobin testing following total shoulder arthroplasty. JSES Int..

[23] Seibold B.T., Quan T., Zhao A.Y., Parel P.M., Mikula J.D., Mun F., Srikumaran U., Zimmer Z.R. (2025). Higher modified frailty index score is associated with 30-day postoperative complications following revision total shoulder arthroplasty. Shoulder Elb..

[24] Lee D., Lee R., Fassihi S.C., Stadecker M., Heyer J.H., Stake S., Rakoczy K., Rodenhouse T., Pandarinath R. (2022). Risk Factors for Blood Transfusions in Primary Anatomic and Reverse Total Shoulder Arthroplasty for Osteoarthritis. Iowa Orthop. J..

[25] von Elm E., Altman D.G., Egger M., Pocock S.J., Gøtzsche P.C., Vandenbroucke J.P., STROBE Initiative (2007). The Strengthening the Reporting of Observational Studies in Epidemiology (STROBE) Statement: Guidelines for reporting observational studies. Ann. Intern. Med..

[26] Kizilkurt T., Ozkaya M., Balli M., Demirel M., Asik M. (2025). The Effect of Preoperative Intravenous Iron Supplementation on Mortality and Blood Transfusion Requirements in Elderly Patients Undergoing Hip Fracture Surgery: A Prospective Randomized Controlled Trial. J. Clin. Med..

[27] De Angelis A., Minelli M., Della Rocca F., Pagot E., Martorelli F., Simili V., De Grandis C.E., Scardino M. (2026). Patient Blood Management in Total Hip and Knee Arthroplasty Before, During, and After the COVID-19 Pandemic: A Single-Center Retrospective Cohort Study. Medicina.

[28] Kashanchi K.I., Nazemi A.K., Komatsu D.E., Wang E.D. (2021). The Impact of Preoperative Anemia on Complications After Total Shoulder Arthroplasty. JAAOS Glob. Res. Rev..

[29] Berk A.N., Hysong A.A., Kahan J.B., Ifarraguerri A.M., Trofa D.P., Hamid N., Rao A.J., Saltzman B.M. (2024). The efficacy of tranexamic acid in primary anatomic and reverse total shoulder arthroplasty: A systematic review and meta-analysis of level I randomized controlled trials. Shoulder Elb..

[30] Liu X., Liu M., Yang Y., Yue C., Tang Y., Xie D., Gao Q., Wu X., Guo J. (2026). Risk prediction models for blood transfusion in patients undergoing total hip and knee arthroplasty: A systematic review and meta-analysis. Ann. Med..

[31] Chen J., Zhong X., Zhai Y., Zhao C., Lan J., Chen L., Xia Z. (2025). Clinical prediction models for postoperative blood transfusion after total knee arthroplasty: A systematic review and meta-analysis. BMC Musculoskelet. Disord..

[32] Ramadanov N., Fuchs D., Heinz M., Prill R., Becker R. (2026). Perioperative Factors Associated With Allogeneic Blood Transfusion in Elective THA. Orthop. Surg..

[33] Garcia-Maya B., Morais S., Diez-Sebastian J., Antuña S., Barco R. (2023). Drain use can be avoided in reverse shoulder arthroplasty. Injury.

[34] Yamaguchi Y., Urita A., Matsui Y., Tomizawa K., Iwasaki N., Taneichi H. (2025). Effect of topical tranexamic acid administration on postoperative blood loss and hematologic parameters after reverse shoulder arthroplasty. J. Orthop. Surg..

[35] Ramamurti P., Quan T., Swansen T., Pollard T.G., Stadecker M., Gu A., Doerre T., Zimmer Z.R. (2023). Comparison of 30-day complications between reverse shoulder arthroplasty and open reduction internal fixation for the treatment of proximal humerus fractures. Eur. J. Orthop. Surg. Traumatol..

[36] Rojas J., Srikumaran U., McFarland E.G. (2021). Inconclusive evidence for the efficacy of tranexamic acid in reducing transfusions, postoperative infection or hematoma formation after primary shoulder arthroplasty: A meta-analysis with trial sequential analysis. Shoulder Elb..

[37] Beyth S., Fraind-Maya G., Safran O. (2023). Tranexamic Acid Treatment Reduces Blood Loss After Elective and Semi-Urgent Reverse Total Shoulder Arthroplasty. Geriatr. Orthop. Surg. Rehabil..

[38] Sehgal J., Black N.R., Somerson J.S. (2026). Tranexamic acid utilization remains low in anatomical and reverse total shoulder arthroplasty despite decreased postoperative complications: A propensity-matched analysis. Shoulder Elb..

[39] Arapovic A.E., Galasso L.A., Hamid H.S., Childers K., Tran H.N., Wiater J.M. (2026). Risk factors for postoperative anemia and blood transfusion in primary anatomic and reverse total shoulder arthroplasty. JSES Int..

[40] Brown A.N., Yendluri A., Lawrence K.W., Cordero J.K., Moucha C.S., Hayden B.L., Parisien R.L. (2024). The Statistical Fragility of Tranexamic Acid Use in the Orthopaedic Surgery Literature: A Systematic Review of Randomized Controlled Trials. J. Am. Acad. Orthop. Surg..

[41] Fink J., Norceide D.J., Kahlon A., Nguyen K.T., Wu K.A., Mai E., Yendluri A., Brown E.L., Kelly J.D., Li X. (2026). Systematic reviews on tranexamic acid in shoulder arthroplasty frequently exhibit reporting bias. JSES Rev. Rep. Tech..

[42] Xiao C., Liyin H., Sheng C. (2026). Effectiveness and Safety of Oxidized Regenerated Cellulose for Hemostasis in Joint Arthroplasty: A Systematic Review and Meta-Analysis. Clin. Appl. Thromb./Hemost..

[43] Belay E.S., O’Donnell J., Flamant E., Hinton Z., Klifto C.S., Anakwenze O. (2021). Intravenous tranexamic acid vs. topical thrombin in total shoulder arthroplasty: A comparative study. J. Shoulder Elb. Surg..

[44] Kelly M., Turcotte J., Fowler M.B., West M., Lashgari C., Gelfand J. (2022). Impact of tranexamic acid on clinical and hematologic outcomes following total shoulder arthroplasty. Shoulder Elb..

[45] Laungani D., Porto J.R., Haase L., Smith K., Chen R., Gillespie R. (2024). Tranexamic Acid in Total Shoulder Arthroplasty: A Scoping Review of Current Practices and Future Directions. JBJS Rev..

[46] Garcia-Maya B., Morais S., Diez-Sebastian J., Antuña S., Barco R. (2023). The efficacy of topical tranexamic acid in reverse shoulder arthroplasty. Rev. Esp. Cir. Ortop. Traumatol..

[47] Min J.K., Zhang Q.H., Li H.D., Li H., Guo P. (2016). The Efficacy of Bipolar Sealer on Blood Loss in Primary Total Hip Arthroplasty: A Meta-Analysis. Medicine.

[48] Chen X., Yang W., Wang X. (2019). Is bipolar sealer superior than standard electrocautery for blood loss control after primary total knee arthroplasty: A meta-analysis. Medicine.

[49] Chen Z. (2026). Review of the influence of anaemia on the effect of total hip arthroplasty, and the prognosis of patients: Recent advances and future directions. Ann. Med..

[50] Schmerler J., Harris A.B., Hegde V., Oni J.K., Khanuja H.S. (2024). Over the Past Decade, Preoperative Anemia Has Become a Greater Predictor of Transfusions After Total Knee Arthroplasty. J. Arthroplast..

[51] Meckstroth S.H., Chapple A.G., Dasa V., Krause P.C., Leslie L.J., Jones D.D. (2024). Disparities Associated With Total Joint Arthroplasty Transfusion Rates. J. Arthroplast..

[52] Burns K.A., Robbins L.M., LeMarr A.R., Morton D.J., Wilson M.L. (2024). Chronic kidney disease increases cost of care and readmission risk after shoulder arthroplasty. J. Shoulder Elb. Surg..

[53] Turner A.C., Jones H.B., Serbin P.A., Sambandam S.M. (2024). The Impact of Preoperative Co-morbidities on Blood Transfusion Requirements Following Reverse Total Shoulder Arthroplasty. Arch. Bone Jt. Surg..

[54] Garcia-Casanovas A., Bisbe E., Vizoso A., Sarsanedas E., Garcia-Altes A., Colomina M.J., Barquero M., Basora M., Maturity Assessment Model for Patient Blood Management (MAPBM) Working Group (2025). Association between Adherence to Patient Blood Management Recommendations and Postoperative Complications in Hip and Knee Arthroplasty. Anesthesiology.

[55] Sezgin E.A., Budin M., Aydın E., Dasci M.F., Gehrke T., Citak M. (2025). Blood transfusion trends and risk factors in primary and revision shoulder arthroplasty: A single centre analysis. Int. Orthop..

[56] Hertling S., Schleußner E., Loos F.M., Eckhardt N., Kaiser M., Graul I. (2024). Sex differences in inflammatory parameters after shoulder arthroplasty and blood loss. Front. Surg..

[57] Areti A., Montanez B., Perake V., Sambandam S.N. (2025). Impact of morbid obesity on postoperative outcomes in reverse total shoulder arthroplasty: A national inpatient sample analysis. J. Orthop..

[58] Tamayo-Velasco Á., Llau J.V., Colomina M.J., de la Varga-Martínez O., Navarro-Pérez R., López-Herrero R., Almoguera-Fernández J., Alonso-Fernández M., Artiaga-Candia M., Becerra-Bolaños Á. (2026). High inappropriate red blood cell transfusion rate despite low overall use: A real-world multicenter study in 43 Spanish hospitals. Front. Med..

[59] Hatta T., Mashiko R., Kawakami J., Matsuzawa G., Ogata Y., Hatta W. (2024). Evolution of Stemless Reverse Shoulder Arthroplasty: Current Indications, Outcomes, and Future Prospects. J. Clin. Med..

