# Supplementary material for: Preoperative Hemoglobin Discriminates Perioperative Transfusion in Reverse Shoulder Arthroplasty: A Single-Center Preliminary Study with Internal Validation and a Candidate Blood-Preparation Threshold
Source: Medicina (Kaunas). 2026 Jul 27;62(8):1455. doi: 10.3390/medicina62081455 (PMC13515453; doi:10.3390/medicina62081455)
Supplement: Supplementary file 1 [file medicina-62-01455-s001.zip › Table S1.pdf]

Table S1. Firth penalized logistic regression—primary and sensitivity models.

Model	Predictor	Adjusted OR	95% CI	p	n (events)
Primary model (preoperative hemoglobin + age + sex)	Preoperative hemoglobin (per +1 g/dL)	0.37	0.21–0.58	<0.001	213 (17)
	Age (per +1 year)	1.21	1.08–1.39	0.002	
	Female sex (vs male)	1.48	0.39–7.15	0.594	
Univariable (preoperative hemoglobin)	Preoperative hemoglobin (per +1 g/dL)	0.37	0.23–0.55	<0.001	213 (17)
Sensitivity: preoperative anemia (binary) + age + sex	Preoperative anemia (WHO, binary)	5.75	1.32–53.98	0.045	213 (17)
	Age (per +1 year)	1.19	1.08–1.34	0.001	
	Female sex (vs male)	1.86	0.56–7.79	0.337	
Sensitivity: exclude the single TXA-exposed patient	Preoperative hemoglobin (per +1 g/dL)	0.37	0.21–0.58	<0.001	212 (17)
	Age (per +1 year)	1.21	1.08–1.39	0.002	
	Female sex (vs male)	1.47	0.38–7.12	0.600	
Sensitivity: + intraoperative blood loss	Preoperative hemoglobin (per +1 g/dL)	0.37	0.21–0.59	<0.001	213 (17)
	Age (per +1 year)	1.21	1.07–1.38	0.003	
	Female sex (vs male)	1.40	0.37–6.66	0.644	

	Intraoperative blood loss (per +1 mL)	1.00	1.00–1.00	0.179	
Sensitivity: + body mass index	Preoperative hemoglobin (per +1 g/dL)	0.34	0.19–0.55	<0.001	209 (16)
	Age (per +1 year)	1.19	1.06–1.37	0.005	
	Female sex (vs male)	1.64	0.42–8.43	0.510	
	Body mass index (per +1 kg/m²)	1.04	0.87–1.24	0.682	

All models fitted by Firth penalized logistic regression (Jeffreys-prior bias correction) for the rare-event outcome (17 events; 16 in the body-mass-index model, which had four missing values). The preoperative-hemoglobin effect is stable across every parameterization. OR, odds ratio; CI, confidence interval; TXA, tranexamic acid; WHO, World Health Organization.
