# Supplementary material for: Preoperative Hemoglobin Discriminates Perioperative Transfusion in Reverse Shoulder Arthroplasty: A Single-Center Preliminary Study with Internal Validation and a Candidate Blood-Preparation Threshold
Source: Medicina (Kaunas). 2026 Jul 27;62(8):1455. doi: 10.3390/medicina62081455 (PMC13515453; doi:10.3390/medicina62081455)
Supplement: Supplementary file 1 [file medicina-62-01455-s001.zip › Table S2.pdf]

Table S2. Completeness of analytic variables (N = 214).

Variable	n with data	n missing	% complete
Age	214	0	100.0
Sex	214	0	100.0
Body mass index	210	4	98.1
Hypertension	213	1	99.5
Diabetes mellitus	213	1	99.5
Chronic kidney disease	214	0	100.0
Cardiovascular disease	214	0	100.0
Anesthesia duration	214	0	100.0
Preoperative hemoglobin	213	1	99.5
Nadir hemoglobin	213	1	99.5
Preoperative anemia (WHO)	213	1	99.5
Perioperative transfusion (outcome)	214	0	100.0

Hemoglobin-derived variables and the comorbidity fields each lack a single value; the Firth regression and ROC analyses were performed on the 213 procedures with a complete preoperative hemoglobin.
